# A Danshensu-Tetramethylpyrazine Conjugate DT-010 Overcomes Multidrug Resistance in Human Breast Cancer

**DOI:** 10.3389/fphar.2019.00722

**Published:** 2019-06-26

**Authors:** Xinhua Zhou, Anqi Wang, Liang Wang, Jianhua Yin, Li Wang, Lijun Di, Maggie Pui-Man Hoi, Luchen Shan, Xu Wu, Yuqiang Wang

**Affiliations:** ^1^State Key Laboratory of Quality Research in Chinese Medicine and Institute of Chinese Medical Sciences, University of Macau, Macao, China; ^2^PU-UM Innovative Institute of Chinese Medical Sciences, Zhuhai, China; ^3^Institute of Biomedical and Pharmaceutical Sciences, Guangdong University of Technology, Guangzhou, China; ^4^Laboratory of Molecular Pharmacology, Department of Pharmacology, School of Pharmacy, Southwest Medical University, Luzhou, China; ^5^South Sichuan Institute of Translational Medicine, Luzhou, China; ^6^Faculty of Health Sciences, University of Macau, Macao, China; ^7^Institute of New Drug Research, College of Pharmacy, Jinan University, Guangzhou, China

**Keywords:** danshensu, tetramethylpyrazine, breast cancer, resistance, glycolysis, P-glycoprotein

## Abstract

**Background:** We previously demonstrated that a Danshensu-Tetramethylpyrazine conjugate DT-010 enhanced anticancer effect of doxorubicin (Dox) in Dox-sensitive human breast cancer cells, and protected against Dox-induced cardiotoxicity. This work was designed to see whether DT-010 overcomes Dox resistance in resistant human breast cancer cells.

**Methods:** The effects of DT-010, Dox or their combination on cell viability of Dox-resistant human breast cancer MCF-7/ADR cells were conducted using 3-(4,5-Dimethylthiazol-2-yl)-2,5-diphenyltetrazolium bromide (MTT) assay. Apoptosis was examined by flow cytometry after Annexin V-FITC/PI co-staining. Dox accumulation in MCF-7/ADR cells was detected by flow cytometry and fluorescence microscopy. A fluorometric multidrug resistance (MDR) assay kit was used to evaluate the effect of DT-010 on MDR transporter activity. P-glycoprotein (P-gp) expression and activity were analyzed by Western blot and rhodamine 123 (Rh123) efflux assay, respectively. The effects of DT-010 on glycolysis and mitochondrial stress were detected using an Extracellular Flux Analyzer. A Succinate Dehydrogenase Activity Assay kit was used to measure mitochondrial complex II activity.

**Results:** At non-cytotoxic concentrations, DT-010 in combination with Dox led to a significant growth inhibition of MCF-7/ADR cells, suggesting a synergy between DT-010 and Dox to reverse Dox resistance. DT-010 restored Dox-mediated apoptosis and p53 induction in MCF-7/ADR cells. DT-010 increased Dox accumulation in MCF-7/ADR cells *via* inhibiting P-gp activity, but without changing P-gp expression. Further studies showed that DT-010 significantly inhibited glycolysis and mitochondrial function of MCF-7/ADR cells. Mitochondrial complex II activity was inhibited by DT-010 or DT-010/Dox combination, but not by Dox. The DT-010-mediated suppression of metabolic process may render cells more vulnerable to Dox treatment and thus result in enhanced efficacy.

**Conclusions:** The results indicate that DT-010 overcomes Dox resistance in human breast cancer cells through a dual action *via* simultaneously inhibiting P-gp-mediated drug efflux and influencing metabolic process.

## Introduction

Breast cancer is the most frequent cancer and the leading cause of cancer-associated death among women worldwide (Siegel et al., 2018). Progress in early diagnosis and improved therapeutic strategies has greatly prolonged overall survival of patients with breast cancer. Chemotherapy, hormonal therapy, and targeted therapy are commonly used for the treatment of breast cancer. However, although patients are responsive to initial drug treatment, progressive disease occurs invariably, and the response rate becomes decreased due to the occurrence of multidrug resistance (MDR). The main mechanisms of MDR include the increased drug efflux, deregulated apoptosis or survival, alteration of drug targets, as well as adapted metabolic reprogramming (Kesharwani et al., 2018; Zhang et al., 2018; Zhong et al., 2018; Zhou et al., 2019). In most cases, multiple MDR impairments may simultaneously occur, which gives rise to challenge in successful therapies in cancer. Strategies to inhibit or bypass MDR processes are thus highly advocated in cancer therapy.

**Figure 1:**
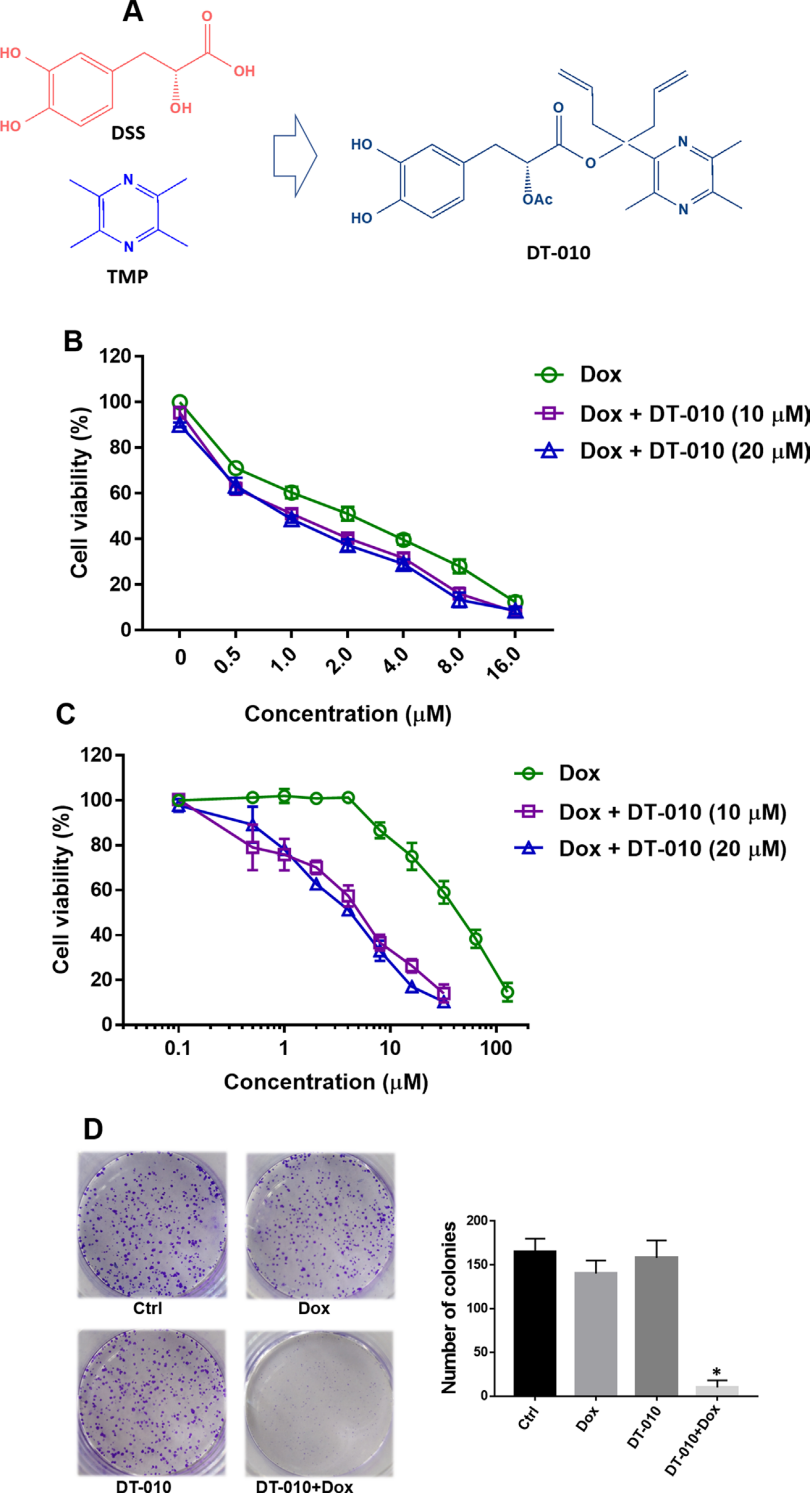
**(A)** Structures of Danshensu (DSS), tetramethylpyrazine (TMP), and DT-010. A bulky hindrance was introduced on the linker between DSS and TMP. **(B** and **C)** Cell viability of MCF-7 and MCF-7/ADR cells after treatment of Dox, DT-010, or their combinations for 24 h. Cell viability was determined using 3-(4,5-Dimethylthiazol-2-yl)-2,5-diphenyltetrazolium bromide (MTT) assay. Results are displayed as mean ± SD. **(D)** Colony formation of MCF-7/ADR cells after treatment of Dox (2 μM), DT-010 (10 μM) or their combinations for 7 days. Cells were stained with 0.1% crystal violet, with colony number counted. Results are displayed as mean ± SD. *p < 0.05, compared to control group.

Natural products are rich sources for discovery of novel lead compounds to design MDR reversal agents (Wen et al., 2018). Danshensu (DSS) and tetramethylpyrazine (TMP) are major bioactive ingredients from the Chinese herbs *Salvia miltiorrhiza* Bge. (Danshen) and *Ligusticum Wallichii* Franch (Chuanxiong), respectively. DSS was reported to possess cardioprotective effect in myocardial ischemia/reperfusion injury both *in vitro* and *in vivo* (Li, 2007; Yin et al., 2013). TMP showed significant anti-oxidant, anti-inflammatory, and neuroprotective activities (Ozaki, 1992; Zhang et al., 2003; Kao et al., 2006) and was able to reverse MDR in human breast cancer cells *via* regulating P-glycoprotein (P-gp) (Zhang et al., 2012). We recently demonstrated that a novel conjugate of DSS and TMP, DT-010 (**Figure 1A**), exerted more potent antitumor activity than parent compounds or their combination, and enhanced the chemotherapeutic efficacy of doxorubicin (Dox) in Dox-sensitive human breast cancer cells by inhibition of the mitochondrial respiratory chain and the glycolysis pathway (Wang et al., 2016a; Wang et al., 2016b). Furthermore, DT-010 protected against Dox-induced cardiotoxicity *in vivo* (Tang et al., 2017; Zhang et al., 2017). To further potentiate DT-010 to be used as an adjuvant in Dox-based chemotherapy, in the present study, we aim to evaluate whether DT-010 can overcome Dox resistance in human breast MDR cancer cells. The results suggest that DT-010 at non-cytotoxic concentrations reverses MDR of Dox in human breast cancer MCF-7/ADR cells potentially through a dual action. This study would provide evidences for the use of DT-010 in combinational therapy to overcome Dox resistance in breast cancer.

## Materials and Methods

### Chemicals and Reagents

DT-010 (purity > 98%) was synthesized as previously reported (Wang et al., 2016a). Dox (Purity > 99%) was purchased from Dalian Meilun Biology Technology Co., Ltd. (Liaoning, China). Dulbecco’s Modified Eagle Medium (DMEM), fetal bovine serum (FBS), penicillin-streptomycin, 0.25% (w/v) trypsin/ethylenediaminetetraacetic acid, and phosphate-buffered saline (PBS) were obtained from Life Technologies (Grand Island, USA). Paraformaldehyde (PFA) and 3-(4,5-dimethylthiazol-2-yl)-2,5-diphenyltetrazolium bromide (MTT) were supplied by Sigma-Aldrich (St. Louis,​ MO, USA). Rhodamine 123 (Rh123) was from Solarbio (Beijing, China). PSC-833 was obtained from MCE (China). Trizol reagent was from Takara (Dalian, China). FastKing RT reagent kit was brought from Tiangen (Beijing, China). SYBR Green Real-Time PCR kit was from Life technologies (USA). Annexin V-FITC/PI Apoptosis Staining Detection Kit (ab14085) was from Abcam (Cambridge, MA, USA). The fluorometric MDR assay kit was from Abcam (Cambridge, UK). An XF Cell Mito Stress Test Kit and an XF Glycolysis Stress Test Kit were purchased from Seahorse Bioscience (USA). Succinate Dehydrogenase Activity Assay kit was obtained from BioVision (USA). The primary antibodies against poly ADP-ribose polymerase (PARP), P-gp, p53, Bax, and glyceraldehyde-3-phospate dehydrogenase (GAPDH) and secondary antibodies were purchased from Cell Signaling Technology (Danvers, MA, USA). All reagent water used was prepared with a Milli-Q apparatus (Millipore Corporation, Darmstadt, Germany). All other chemicals were of the highest purity commercially available.

### Cell Lines and Cell Culture

Human breast cancer cell line MCF-7 were obtained from the American Type Culture Collection (ATCC, Manassas, VA, USA). Dox-resistant MCF-7/ADR cells were selected in stepwise increasing concentrations of Dox as previously described (Wang et al., 2014b). MCF-7/ADR cells were maintained with 1 μM Dox every three passages to keep cells resistant to the drug. Cells were cultured in DMEM with 10% (v/v) FBS and antibiotics containing 100 U/ml penicillin and 100 μg/ml streptomycin and maintained at 37°C in a 5% CO_2_ atmosphere.

### Cell Viability Assay

Exponentially growing cells were seeded into 96-well plates at a density of 3,000 cells/well in 100-μl medium and allowed to attach overnight. Cells were treated with designated drugs or drug combinations for 24 h. Cell viability was assessed using MTT method as previously described (Zhong et al., 2018). The concentration for half maximum inhibitory effect (IC_50_) was calculated by GraphPad Prism 5. Resistance factor (fold of resistance) was calculated as the IC_50_ value of Dox in MCF-7/ADR cells divided by IC_50_ value of Dox in MCF-7 cells. Synergy between DT-010 and Dox was assessed by calculating combination index (CI) based on cell viability results using CompuSyn software. A CI < 0.9 is considered synergistic effect, CI around 0.9 to 1.1 was additive effect, and CI > 1.1 was antagonistic effect.

### Colony Formation Assay

Exponentially growing cells were seeded into six-well plates at a density of 500 cells/well in 2 ml medium and allowed to attach overnight. Cells were treated with designated drugs or drug combinations for 7 days. Cell colonies were stained with 0.1% crystal violet at room temperature for 0.5 h, then rinsed with tap water. The number of colonies (larger than 50 µm in diameter) was counted microscopically.

### Apoptosis Assay

Apoptosis was detected using an Annexin V-FITC/PI detection kit according to the manufacturer’s protocol. Briefly, cells were collected, washed three times with cold PBS and gently suspended in 100-μl binding buffer, followed by staining with Annexin V-FITC (5 μl) and PI (10 μl) solution, incubating for 15 min and analyzing on a flow cytometer (BD FACS Canto™). Triplicated experiments were performed.

### Determination of Intracellular Dox

The intracellular level of Dox was analyzed by flow cytometry and fluorescence microscopy. Briefly, MCF-7 and MCF-7/ADR cells were pretreated with DT-010 for 12 h and then incubated with 2 μM Dox for 4 h, and cells were washed three times with cold PBS. For flow cytometric analysis, cells were collected and analyzed using a flow cytometer (BD FACS Canto™) in FL2 PE channel. Generally, a total of 10,000 cells were collected, amplified, and scaled to generate single parameter histogram. For observation by fluorescence microscopy, cells were fixed with 4% PFA, washed with PBS, stained with Hoechst 33342 (1 μg/ml), and imaged using an Incell Analyzer 2000 (GE Healthcare Life Sciences, USA). Each condition was performed in triplicate.

### MDR Transporter Activity Assay

MDR transporter activity was detected based on a fluorometric MDR assay kit according to the manufacturer’s instruction. Briefly, cells (1.0 × 10^4^ cells/well) were seeded into 96-well flat clear-bottom black-wall microplates and allowed to attach for 24 h. The cells were treated with DT-010 (5, 10, and 20 μM) for 1 h. Then 100 μl MDR dye-loading solution was added into each well and incubated at 37°C for another 1 h in dark. Intracellular fluorescence was determined using SpectraMax M5 microplate reader with excitation wavelength of 490 nm and emission wavelength of 525 nm. Experiments were performed in triplicate.

### P-gp Efflux Assay

The P-gp substrate Rh123 efflux assay was conducted as previously reported (Wang et al., 2019). Briefly, cells were incubated with Rh123, Rh123 with the DT-010 or Rh123 with PSC-833 (P-gp inhibitor as positive control) at 37°C for 30 min. After a Rh123-free efflux for 1 h, fluorescence retention in the cells was measured by flow cytometry.

### Western Blot

Cells were lysed with radio-immunoprecipitation assay lysis buffer containing 1% protease inhibitor. Cell extracts were resolved by sodium dodecyl sulfate polyacrylamide gel electrophoresis (SDS-PAGE) gel and transferred onto a nitrocellulose membrane. After blocking with 5% nonfat milk in Tris-buffered saline (50 mM Tris-HCl, pH 7.5, 150 mM NaCl) containing 0.1% Tween 20, the membranes were probed with the corresponding primary antibodies. Following incubation with anti-mouse or anti-rabbit IgG horseradish peroxidase conjugate, protein bands were visualized using enhanced chemoluminescence blotting detection reagents (Clarity, Bio-Rad).

### Quantitative PCR

Total RNA isolation from cells was conducted using Trizol reagent (Ambion, Life Technologies) following the manufacturer’s protocol. Reverse transcription PCR was performed using FastKing RT reagent kit. Quantitative PCR (qPCR) analysis was performed in an CFX96 Touch Real Time PCR System (BioRad) using SYBR Green Real Time PCR kit. Primers for qPCR reactions are *GAPDH* (forward, 5′-GTCAAGGCTGAGAACGGGAA-3′; reverse, 5′-AAATGAGCCCCAGCCTTCTC-3′), *ABCB1* (forward, 5′-CCCATCATTGCAATAGCAGG-3′; reverse, 5′-TGTTCAAACTTCTGCTCCTGA-3′), *ABCC1* (forward, 5′-ATGTCACGTGGAATACCAGC-3′; reverse, 5-GAAGACTGAACTCCCTTCCT-3), *ABCG2* (forward, 5′-AGATGGGTTTCCAAGCGTTCAT-3′; reverse, 5′-CCAGTCCCAGTACGACTGTGACA-3′) and *SLC22A16* (forward, 5′-GCCCTCCTGAGTGGAGTGTTAA-3′; reverse, 5′-TTTCATTCTCTGACTCCAGTTTTGC-3′).

### Measurement of Metabolic Parameters

The glycolysis and mitochondrial stress tests were determined by an extracellular flux analyzer as previously described (Wang et al., 2016a). Briefly, cells were seeded into Seahorse 24-well tissue culture plates for 24 h. After 12 h of DT-010 (20 μM) treatment, the changes of extracellular acidification rate (ECAR) and oxygen consumption rate (OCR) values were recorded after the addition of metabolic reagents included in XF Glycolysis Stress Test Kit and XF Cell Mito Stress Test Kit, respectively. All measurements were normalized to the protein contents in each well. The levels of basal glycolysis, glycolytic capacity, and glycolytic reserve were calculated based on ECAR data, whereas the levels of basal respiration, ATP production, and maximal respiration were calculated based on OCR data.

### Measurement of Mitochondrial Complex II Activity

The mitochondrial complex II activity of MCF-7/ADR cells was determined by Succinate Dehydrogenase Activity Assay kit (BioVision). Cells seeded into six-well plates were treated with DT-010, Dox or their combination for 12 h, which were collected for detection of succinate dehydrogenase (SDH) activity according to the manufacturer’s instruction.

### Statistical Analysis

All results were expressed as mean ± SD. Statistical analysis was performed using GraphPad Prism 5 software (GraphPad Software, San Diego, CA, USA). One-way ANOVA followed by Dunnett’s multiple comparisons test was used for statistical comparison among multiple groups, where a *p*-value less than 0.05 is considered of statistical signiﬁcance.

## Results

### DT-010 Overcomes Dox Resistance in MCF-7/ADR Cells

DT-010 is a conjugate of DSS and TMP (**Figure 1A**). In consistent with previous study (Wang et al., 2016b), we showed that DT-010 at 10 and 20 μM showed slight growth-inhibitory effect in Dox-sensitive MCF-7 cells, and DT-010 significantly enhanced Dox sensitivity in MCF-7 cells (**Figure 1B**). The IC_50_ value of Dox in MCF-7 cells was decreased from 1.96 ± 0.1 μM (Dox alone) to 1.14 ± 0.08 μM (Dox plus 10 μM DT-010) and 1.03 ± 0.09 μM (Dox plus 20 μM DT-010). However, the effect was not synergistic, as the CI value calculated was around 1.0 indicating an addictive effect.

As shown in **Figure 1C**, DT-010 did not induce cytotoxicity in MCF-7/ADR cells at a concentration up to 20 μM. Dox was very resistant to MCF-7/ADR cells, with an IC_50 _value of 40.7 ± 2.3 μM (20.7-fold of resistance compared to that of MCF-7 cells). Interestingly, cell viability assay showed that, at non-toxic concentrations of 10 and 20 μM, DT-010 remarkably sensitized MCF-7/ADR cells to Dox (**Figure 1C**). In the presence of DT-010 at 10 and 20 μM, the IC_50_ of Dox was 4.61 ± 1.02 μM and 3.70 ± 0.58 μM, respectively, with resistance factors (fold of resistance) reduced to 2.35 and 1.87. Moreover, the CI values calculated were less than 0.1. Similarly, either DT-010 (10 μM) or Dox (0.5 μM) could not inhibit colony formation of MCF-7/ADR cells, while their combination resulted in almost complete suppression of growth (**Figure 1D**). The results suggest a synergistic effect between DT-010 and Dox which is specific in MCF-7/ADR cells.

### Combinational Treatment of DT-010 with Dox Evokes Apoptosis in MCF-7/ADR Cells

**Figure 2:**
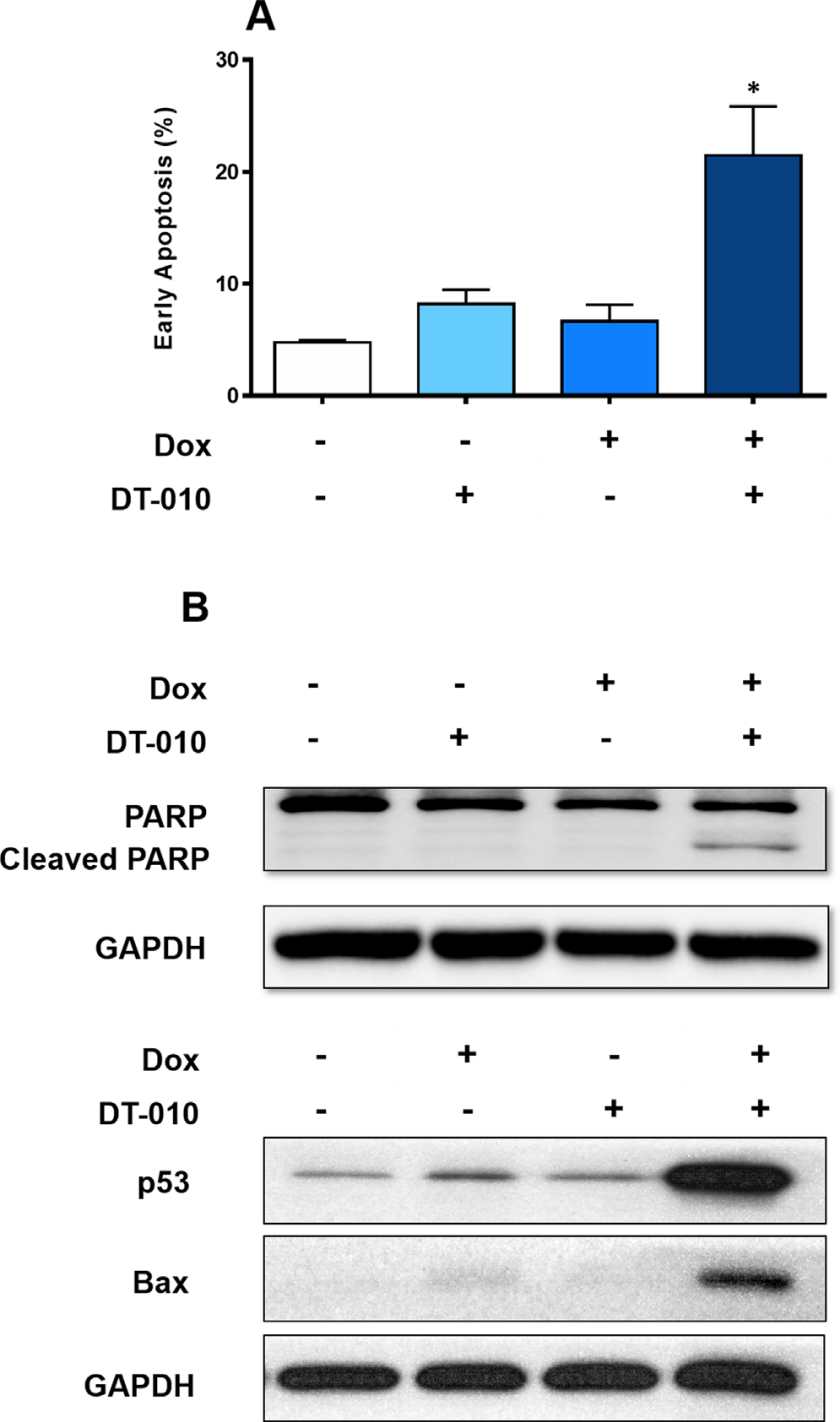
DT-010 restored Dox-mediated apoptosis in MCF-7/ADR cells. **(A)** MCF-7/ADR cells were treated with Dox (2 μM), DT-010 (20 μM) or the combination for 24 h, followed by flow cytometric analysis after PI/Annexin-V staining. Early apoptosis rate was calculated based on population of PI−/Annexin-V+ cells. Experiments were performed in triplicate. Results were displayed as mean ± SD. *p < 0.05, compared with control or other groups. **(B)** Western blot was performed using cell lysate of MCF-7/ADR cells after treatment of Dox (2 μM), DT-010 (20 μM) or the combination for 24 h. Anti-GAPDH was used as loading control.

It is indicated that treatment of either Dox (2 μM) or DT-010 (20 μM) alone for 24 h did not induce apoptosis in MCF-7/ADR cells (**Figure 2A**). Remarkably, their combination resulted in a significant increase of early apoptotic cell population (PI negative/Annexin V positive cells) to nearly 21%. Furthermore, PARP cleavage was observed upon cotreatment of Dox and DT-010 (**Figure 2B**).

Dox is known to activate p53 pathway to trigger apoptosis. We showed that Dox alone slightly increased p53 expression in MCF-7/ADR cells, and DT-010 had no effect. Notably, a substantial upregulation of p53 and its downstream target Bax was observed using a combination of DT-010 and Dox (**Figure 2B**). The results suggest that DT-010 restores Dox-induced activation of p53 pathway in MCF-7/ADR cells.

### DT-010 Increases Dox Accumulation in MCF-7/ADR Cells Through Inhibiting P-gp

**Figure 3:**
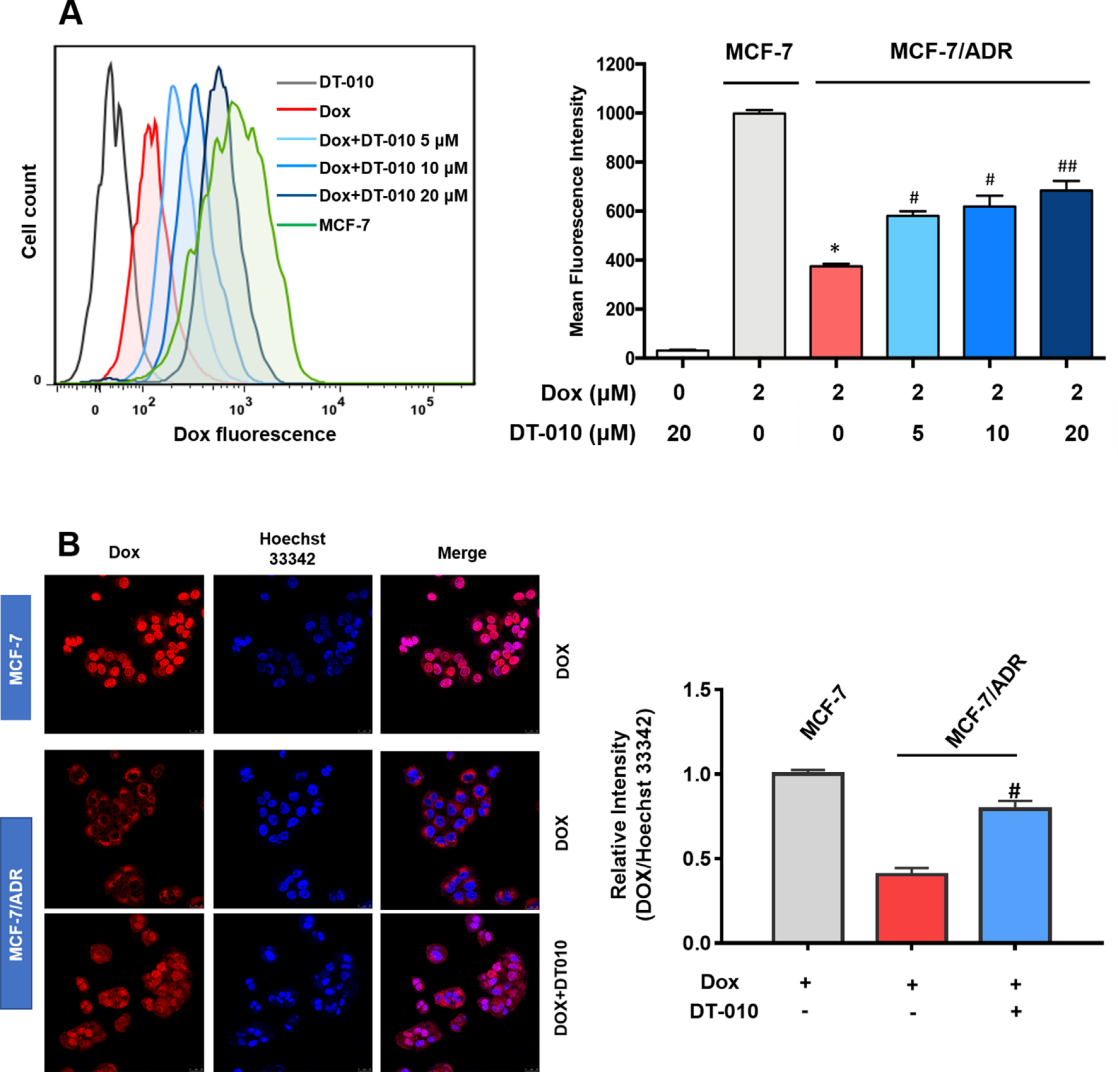
DT-010 increased Dox concentration in MCF-7/ADR cells. MCF-7/ADR cells were pretreated with DT-010 (0, 5, 10, and 20 μM) for 12 h, followed by incubated with Dox (2 μM) for another 4 h. The intracellular concentration of Dox was analyzed by flow cytometry **(A)** and observed using Incell Analyzer 2000 (GE Healthcare) **(B)**. MCF-7 cells treated with Dox (2 μM) for 4 h were used for a comparison. Mean fluorescence intensity were recorded in flow cytometry assay. Relative fluorescence intensity (nuclear area) of Dox over Hoechst 33342 was calculated by ImageJ. Data are expressed as mean ± SD. *p < 0.05, compared with MCF-7 cells. ^#^p < 0.05, ^##^p < 0.001, compared with Dox alone in MCF-7/ADR cells.

Further study was performed to see whether and how DT-010 influences Dox accumulation in MCF-7/ADR cells. As shown in **Figure 3A**, under the same condition, the level of Dox in MCF-7/ADR cells was significantly lower than that in the MCF-7 cells. The addition of DT-010 at 5, 10, and 20 μM significantly elevated fluorescence intensity in MCF-7/ADR cells, indicating an increase of cellular uptake of Dox. The accumulation of Dox was also demonstrated by fluorescence microscopy (**Figure 3B**). Notably, we observed a significant increase of accumulation of Dox in nuclear area of MCF-7/ADR cells, as reflected by the relative intensity of Dox and Hoechst 33342 fluorescence.

**Figure 4:**
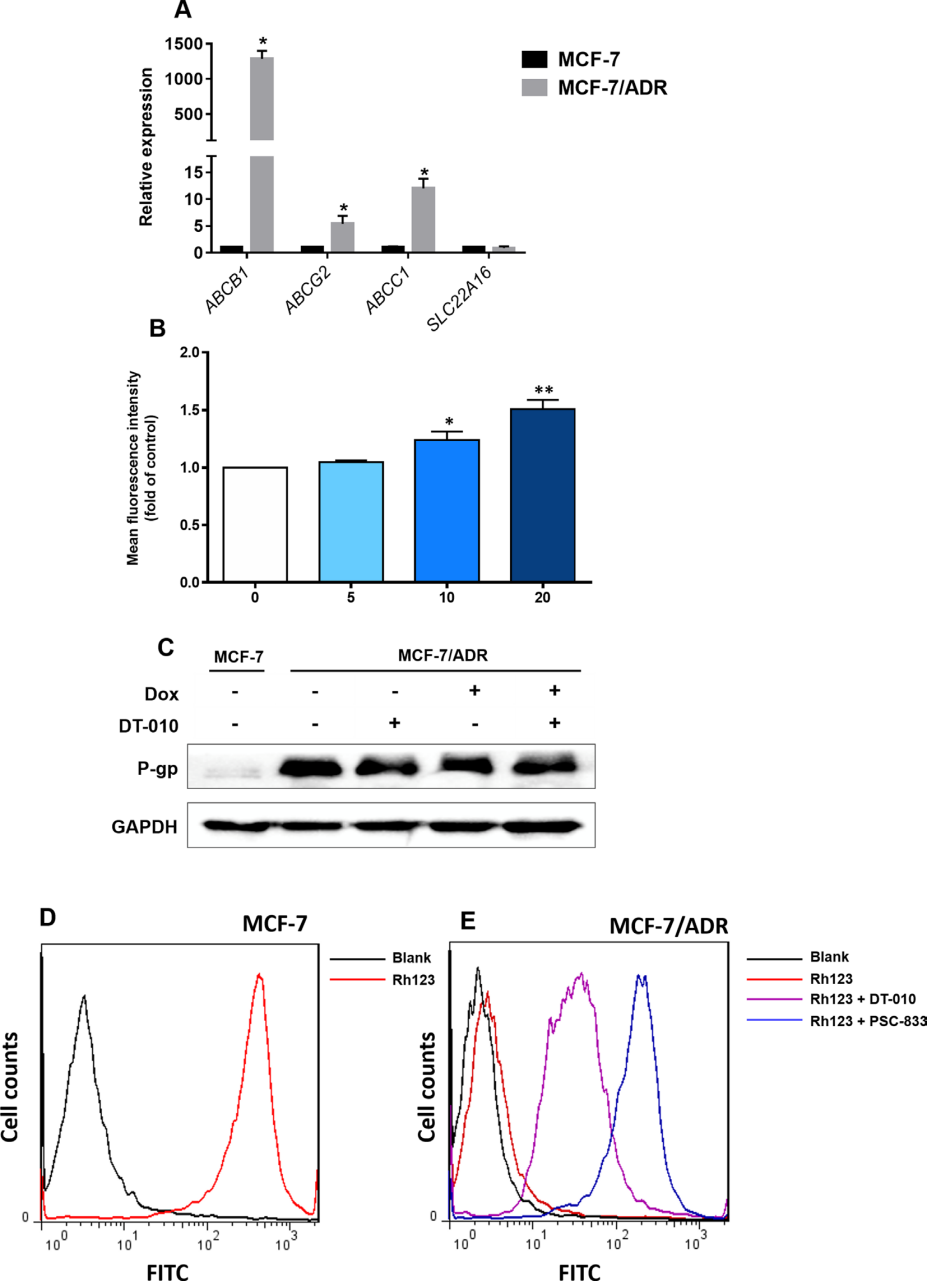
Effects of DT-010 on expression and activity of P-gp. **(A)** Relative messenger RNA expression of ABCB1, ABCC1, ABCG2, and SLC22A16 in MCF-7 and MCF-7/ADR cells. Data are expressed as mean ± SD. *p < 0.05, compared with MCF-7 cells. **(B)** Multidrug resistance (MDR) transporter activity was detected using a fluorometric MDR assay kit. MCF-7/ADR cells were treated with DT-010 (5, 10, and 20 μM) for 1 h, followed by the addition of 100 μl MDR dye-loading solution and incubated at 37°C for another 1 h. Data are expressed as mean ± SD. *p < 0.05, or **p < 0.01, compared with control. **(C)** DT-010 (20 μM), Dox (2 μM), or their combination did not affect P-gp expression in MCF-7/ADR cells. Western blot was performed, with anti-GAPDH as loading control. **(D** and **E)** The effect of DT-010 on P-gp substrate efflux activity was assessed. MCF-7/ADR cells (5.0 × 10^4^/well) were incubated with the P-gp-specific fluorescent substrate Rh123 at 0.5 μg/ml with or without DT-010 (20 μM) for 1 h, then washed twice with ice-cold phosphate-buffered saline (PBS), and incubated in Rh123-free medium at 37°C for additional 1 h with or without DT-010. Cells were analyzed on flow cytometry to detect Rh123 fluorescence. The PSC-833 (0.1 μM) was used as a positive control. MCF-7 cells were used for confirmation of overexpression of P-gp (increased Rh123 efflux) in MCF-7/ADR cells.

Efflux transporters (e.g., P-gp/ABCB1, ABCC1, and ABCG2) and influx transporters (e.g., SLC22A16) are involved in Dox resistance in cancer cells (Thorn et al., 2011; Wang et al., 2017). We demonstrated that MCF-7/ADR cells was overexpressed with *ABCB1*, *ABCC1,* and *ABCG2*, but not *SLC22A16* (**Figure 4A**). Compared to MCF-7 cells, the relative expression of *ABCB1*, *ABCC1*, and *ABCG2* was 1,287-, 12.0-, and 5.4-fold higher in MCF-7/ADR cells, respectively (**Figure 4A**). To see whether DT-010 affects these efflux transporters, the fluorometric MDR assay showed that DT-010 at 10 and 20 μM significantly stimulated activity of MDR transporters (**Figure 4B**). Since P-gp is the most expressed efflux transporter in MCF-7/ADR cells, we firstly focused on whether DT-010 influences P-gp expression and function. It was indicated that DT-010 or Dox alone, or their combination did not affect P-gp expression in MCF-7/ADR cells (**Figure 4C**). P-gp function was monitored using an Rh123 efflux assay. Rh123 as a known substrate of P-gp was accumulated in MCF-7 cells but not in MCF-7/ADR cells (**Figure 4D and E**). The specific P-gp inhibitor PSC-833 (0.1 μM) completely restored Rh123 accumulation in MCF-7/ADR cells, suggesting P-gp is responsible for Rh123 efflux. Notably, DT-010 also remarkably promoted entry of Rh123 into MCF-7/ADR cells, but to a lessor level compared to that observed using PSC-833 (**Figure 4E**). The results suggest that DT-010 increases Dox accumulation in Dox-resistant cells which is partially due to P-gp inhibition.

### DT-010 Inhibits Glycolytic Pathway in MCF-7/ADR Cells

**Figure 5:**
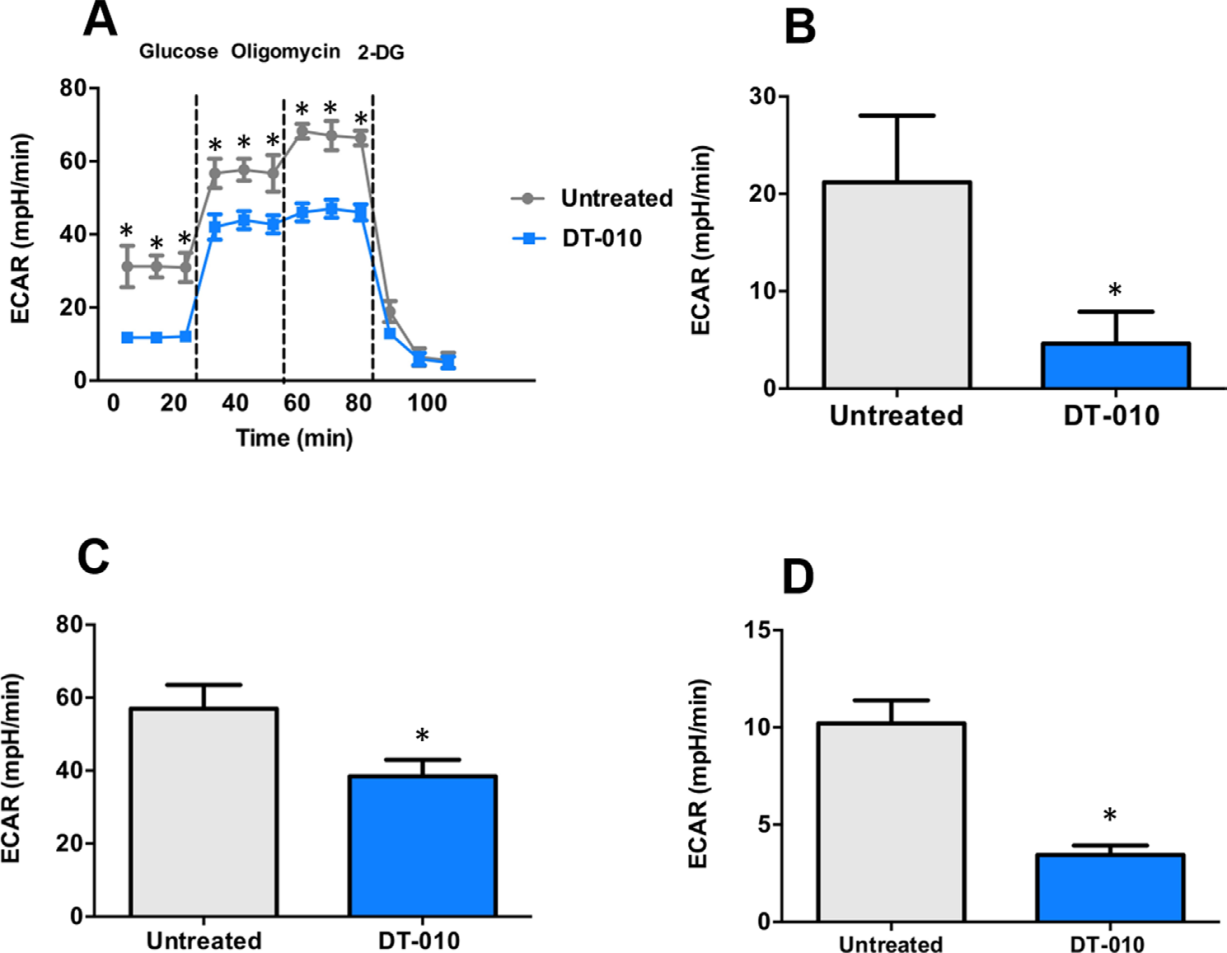
DT-010 inhibited glycolytic pathway of MCF-7/ADR cells. The extracellular acidification rate (ECAR) was measured with XF24 extracellular flux analyzer after 12 h of DT-010 treatment **(A)**. The basal glycolysis **(B)**, glycolytic capacity **(C)**, and glycolytic reserve **(D)** of MCF-7/ADR cells were calculated. *p < 0.05, compared with DT-010-treated group.

The effects of DT-010 on the glycolytic pathway of MCF-7/ADR cells were investigated. The key parameters of glycolysis are the basal glycolysis (ECAR^glucose post injection^ − ECAR^baseline^), glycolytic capacity (ECAR^Oligomycin post injection^ − ECAR^baseline^) and glycolytic reserve (glycolytic capacity − basal glycolysis). As shown in **Figure 5**, the basal glycolysis (**Figure 5B**), glycolytic capacity (**Figure 5C**) and glycolytic reserve (**Figure 5D**) were significantly decreased after DT-010 treatment, showing that DT-010 inhibits glycolytic pathway in MCF-7/ADR cells. Notably, DT-010 also inhibited the basic level of ECAR which is referred as the non-glycolytic process.

### DT-010 Inhibits Mitochondrial Function in MCF-7/ADR Cells

**Figure 6:**
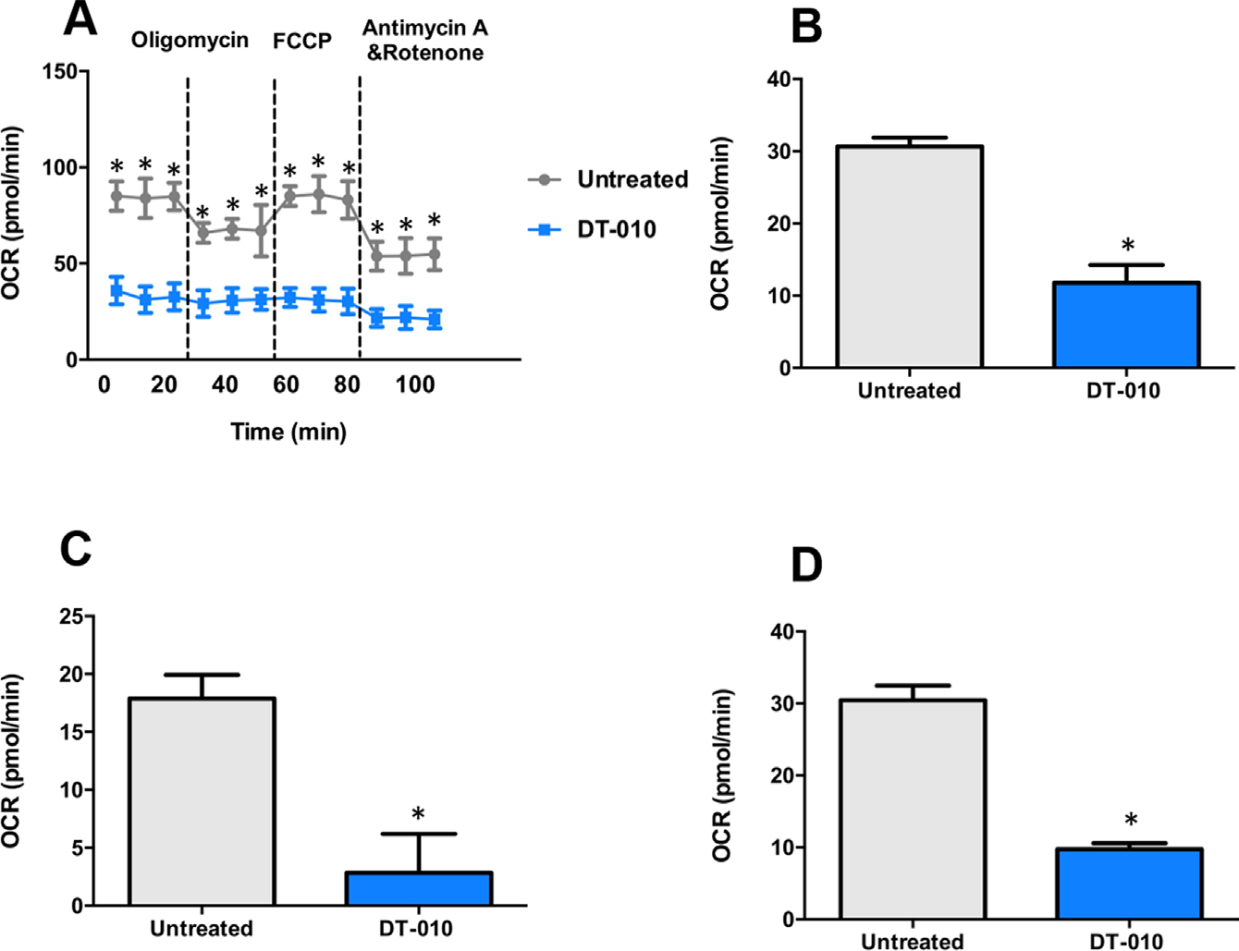
DT-010 inhibited mitochondrial respiration of MCF-7/ADR cells. MCF-7/ADR cells were treated with DT-010 for 12 h, the effects of DT-010 on OCR were monitored with XF24 extracellular flux analyzer **(A)**. The basal respiration **(B)**, ATP production **(C)** and maximal respiration **(D)** of MCF-7/ADR cells were calculated. *p < 0.05, compared with DT-010-treated group.

The effects of DT-010 on the mitochondrial function of MCF-7/ADR cells investigated by the Seahorse XF Extracellular Flux Analyzer. The OCR was monitored (**Figure 6A**) after 12 h of DT-010 treatment. The main parameters of mitochondrial function included basal respiration (OCR^baseline respiration^ − OCR^antimycin A/rotenone post injection^), ATP production (OCR^baseline respiration^ − OCR^oligomycin post injection^) and maximal respiration (OCR^FCCP post injection^ − OCR^antimycin A/rotenone post injection^). We found that the basal respiration of MCF-7/ADR cells (**Figure 6B**), ATP production (**Figure 6C**) and maximal respiration (**Figure 6D**), were significantly inhibited, as compared with the control group. The result indicates that DT-010 suppressed mitochondrial respiration in MCF-7/ADR cells, which may contribute to enhanced vulnerability of cells in response to Dox.

### DT-010 Inhibits Activity of Mitochondrial Complex II in MCF-7/ADR Cells

**Figure 7:**
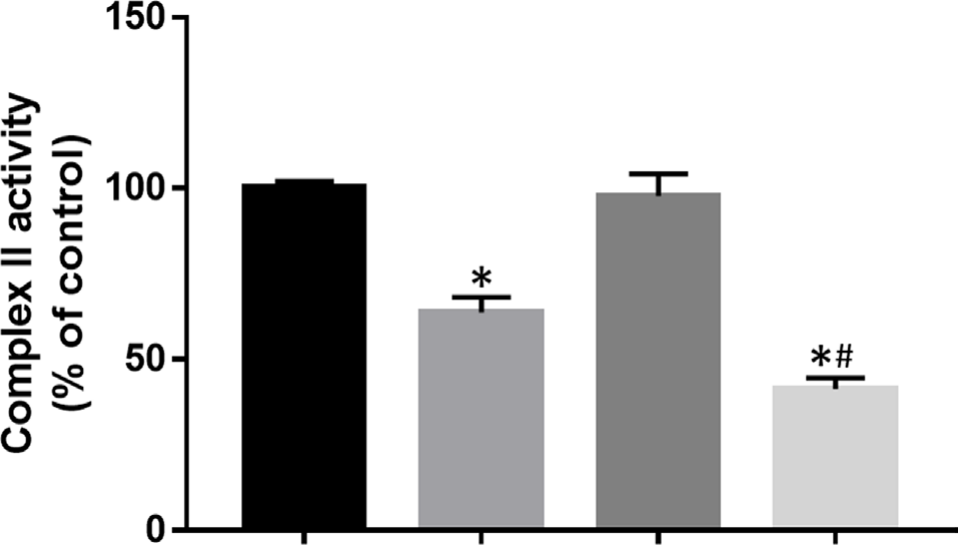
DT-010 inhibited mitochondrial complex II activity in MCF-7/ADR cells. Cells were treated with DT-010 (20 μM), Dox (2 μM), or their combination for 12 h, and complex II activity was measured by Succinate Dehydrogenase Activity Assay kit. *p < 0.05, compared with control. ^#^ *p* < 0.05, compared to DT-010-treated group.

Mitochondrial complex II, also known as SDH, is the only complex which involves in the Krebs cycle and the electron transportation chain. Here, the effect of DT-010 and/or Dox on activity of mitochondrial complex II was further investigated. The result showed that DT-010 significantly inhibited SDH activity while Dox alone had no effect (**Figure 7**). Notably, co-treatment of DT-010 and Dox led to a more suppressive effect on SDH compared to that achieved by DT-010 treatment (**Figure 7**). It is suggested that DT-010-mediated inhibition of glycolysis and mitochondrial function is associated with decreased function of SDH which may be important for synergy between DT-010 and Dox.

## Discussion

Previously, we have shown that DT-010 significantly inhibited cell growth of human breast cancer MCF-7 cells *via* initiating apoptosis through inhibiting mitochondrial respiration and promoting reactive oxygen species (ROS) (Wang et al., 2016b). Moreover, DT-010 enhanced the antitumor effect of Dox (Wang et al., 2016a), and protected from Dox-mediated cardiotoxicity by inhibiting ROS-induced apoptosis and autophagy (Tang et al., 2017; Zhang et al., 2017). Therefore, DT-010 may be developed as a new anticancer drug candidate for breast cancer therapy. Since chemoresistance is very common in breast cancer, it is of primary interests for us to further explore whether DT-010 can sensitize chemotherapy, such as Dox to MDR breast cancer cells. Intriguingly, in the present work, DT-010 was demonstrated to reverse Dox resistance in Dox-resistant MCF-7/ADR cells.

Significant synergy is observed between DT-010 and Dox in MCF-7/ADR cells but not in MCF-7 cells. It is found that both DT-010 and Dox were relatively resistant to MCF-7/ADR cells. Notably, at non-cytotoxic concentrations, DT-010/Dox cotreatment resulted in restored growth inhibition and apoptosis of MCF-7/ADR cells. Thus, the result suggests that DT-010 serves as a chemosensitizer to overcome MDR.

P-gp-mediated efflux transport of chemotherapeutic drugs is one of major causes of MDR in cancers including breast cancer (Wu et al., 2016). TMP is an active constituent isolated from *L. Wallichii* Franch (Chuanxiong), which has previously shown to exhibit inhibitory effect on P-gp expression and function and thus to overcome breast cancer MDR (Zhang et al., 2012). In this work, we demonstrated that DT-010 could restore Dox-mediated apoptosis in MCF-7/ADR cells through increasing intracellular level of Dox potentially *via* influencing P-gp activity. DT-010-mediated P-gp inhibition was further evidenced by the increased uptake of P-gp substrate Rh123 in MCF-7/ADR cells in the presence of DT-010. Notably, the P-gp-inhibitory effect of DT-010 (20 μM) was less than that of the positive control PSC-833 at 0.1 μM. DT-010 did not affect P-gp expression. Since DT-010 at a concentration of 20 μM could not restore Dox level in the MCF-7/ADR cells relative to that in MCF-7 cells, the MDR reversal effect of DT-010 may be only partially due to P-gp inhibition.

Increased evidences have indicated that cancer glycolysis is a novel target for cancer therapy (Ganapathy-Kanniappan and Geschwind, 2013; Alves et al., 2019). Inhibition of glycolysis leads to suppression of cancer cells, reversal of MDR, and enhancement of the efficacy of chemotherapeutic agents. Glycolysis inhibitors, such as 2-DG, significantly reduced ATP levels in cancer cells and showed anticancer effects both *in vitro* and *in vivo* (Pelicano et al., 2006). Treatment with an mTORC1/2 inhibitor AZD2014 and 2-DG synergistically mediated the inhibition of cell growth and apoptosis in non-small cell lung cancer cells (Jiang et al., 2015). It is reported that MDR of cancer was associated with enhanced glycolysis, and inhibition of glycolysis increased the sensitivity of MDR cells to anticancer drugs (Bhattacharya et al., 2014; Wang et al., 2014a). Our results showed that DT-010 suppressed basal glycolysis, glycolytic capacity, and glycolytic reserve of MCF-7/ADR cells, along with reduced ATP production and mitochondrial respiration. Thus, DT-010 may reduce the threshold of glycolysis in resistant cancer cells, and render them more susceptible to chemotherapy. This is consistent with our finding on the chemo-sensitive MCF-7 cells where DT-010 enhanced Dox activity (Wang et al., 2016a). It is likely that inhibition of glycolysis by DT-010 is crucial for improving effects of conventional chemotherapeutic drugs, such as Dox. This is also evidenced by the fact that DT-010 but not Dox inhibits activity of mitochondrial complex II, a key complex critically involved in the metabolic process.

In summary, DT-010 overcomes Dox resistance in MCF-7/ADR cells potentially through a dual action, inhibition of P-gp, and glycolysis.

## Conclusion

In the present study, we show that a DSS-TMP conjugate DT-010 at non-cytotoxic concentrations reverses Dox MDR in resistant human breast cancer MCF-7/ADR cells. The synergistic anticancer effect between Dox and DT-010 is at least partially associated with P-gp inhibition. Moreover, DT-010 significantly inhibits glycolysis, mitochondrial respiration, and mitochondrial complex II activity of MCF-7/ADR cells, which may lead to enhanced vulnerability of cells and thus increased efficacy in response to Dox. Further to our previous findings on anticancer effect of DT-010 in chemo-sensitive breast cancer cells, the present study provides new evidences for using DT-010 as a chemosensitizer in MDR breast cancer cells.

## Author Contributions

XZ, AW, and JY performed experiments. LW, LS, and XW designed the experiments and wrote the manuscript. LW, LD, and MH analyzed the data. XW and YW organized and supervised the study. All authors have read and approved the final manuscript.

## Funding

This work was supported by grants from the National Natural Science Foundation of China (81703807 and 81703509) and the Joint Funds of the Southwest Medical University & Luzhou, Sichuan Province, China (2018LZXNYD-ZK34).

## Conflict of Interest Statement

The authors declare that the research was conducted in the absence of any commercial or financial relationships that could be construed as a potential conflict of interest.
